# Modeling of the Ebola Virus Delta Peptide Reveals a Potential Lytic Sequence Motif

**DOI:** 10.3390/v7010285

**Published:** 2015-01-20

**Authors:** William R. Gallaher, Robert F. Garry

**Affiliations:** 1 Mockingbird Nature Research Group, PO Box 568, Pearl River, LA 70452, USA; 2 Department of Microbiology, Immunology & Parasitology, Louisiana State University Health Sciences Center, 1901 Perdido Street, New Orleans, LA 70112, USA; 3 Department of Microbiology and Immunology, Tulane University Medical Center, 1430 Tulane Avenue, New Orleans, LA 70112, USA; E-Mail: rfgarry@tulane.edu

**Keywords:** Ebola, ebolavirus, filovirus, lysin, viroporin, enterotoxin, ion channel

## Abstract

Filoviruses, such as Ebola and Marburg viruses, cause severe outbreaks of human infection, including the extensive epidemic of Ebola virus disease (EVD) in West Africa in 2014. In the course of examining mutations in the glycoprotein gene associated with 2014 Ebola virus (EBOV) sequences, a differential level of conservation was noted between the soluble form of glycoprotein (sGP) and the full length glycoprotein (GP), which are both encoded by the GP gene via RNA editing. In the region of the proteins encoded after the RNA editing site sGP was more conserved than the overlapping region of GP when compared to a distant outlier species, Tai Forest ebolavirus. Half of the amino acids comprising the “delta peptide”, a 40 amino acid carboxy-terminal fragment of sGP, were identical between otherwise widely divergent species. A lysine-rich amphipathic peptide motif was noted at the carboxyl terminus of delta peptide with high structural relatedness to the cytolytic peptide of the non-structural protein 4 (NSP4) of rotavirus. EBOV delta peptide is a candidate viroporin, a cationic pore-forming peptide, and may contribute to EBOV pathogenesis.

## 1. Introduction

Filoviruses, first discovered in 1967, are associated with severe febrile illness in humans and non-human primates [1,2,3]. Native to the middle and southern areas of Africa, in March of 2014, it became apparent in the West African country of Guinea that an outbreak of Ebola Virus Disease (EVD) had begun caused by a virus with similarities to those that had earlier appeared only in Middle Africa [4,5]. Generally, filovirus outbreaks have been brought under control by isolating patients ill with filoviral disease, using intensive barrier nursing care, and tracing contacts of those contracting the disease [6]. EVD has since spread across several West African countries, most prominently in Guinea, Sierra Leone and Liberia, and as of early January 2015 had reached over 20,000 total cases with over 7500 deaths [7]. While a few exported cases from the current ongoing outbreak have been reported and quickly contained in Western countries thus far, the virus constitutes a threat of arriving undetected in locations outside of West Africa [8,9,10].

Filoviruses like EBOV are enveloped viruses with a single-stranded, negative polarity genome of approximately 19 kilobases that must be accompanied by a functional viral RNA polymerase to be infectious. The viral genome codes for seven proteins, which are found in infectious virus particles. In order, there are the genes encoding the major nucleoprotein (NP), a second nucleoprotein VP35, a matrix protein VP40, the glycoprotein (GP), two accessory proteins VP24 and VP30, and finally the largest of the viral proteins L, a multifunctional viral polymerase [11]. In ebolaviruses, but not marburgviruses, the glycoprotein gene is transcribed into two or three separate messenger RNAs that code for three different versions of GP [12,13,14,15]. In Ebola virus (EBOV) the GP versions share the first 295 amino acid residues. RNA editing at a site containing seven uridine residues adds one or more additional adenines into the mRNA sequence shifting the reading frame and thereafter altering the amino acid sequence of the carboxy-terminal region of each version of GP [12,14,16,17,18,19]. Full length GP, which is 676 amino acids in length, is produced from mRNAs containing an additional nontemplated adenosine, and is the form that contains both the GP1 attachment protein and the GP2 fusion/entry protein. Soluble GP (sGP) is produced from the unedited RNA transcript, and is a shorter form of the polypeptide that contains only 364–372 amino acids. Small soluble GP (ssGP) is the product of a mRNA produced with two nontemplated adenosines, and is a truncated version of sGP [16]. The RNA editing of GP messenger RNA was discovered in 1995 [18], and sGP generally characterized in 1998–1999 [20]. Just as full length GP is cleavable to produce GP1 and GP2, so also sGP is cleaved at another site in its unique sequence to generate a smaller form of GP1 alone plus a residual 40 amino acid peptide termed “delta peptide” [21]. sGP is made in profuse amounts, and secreted after being proteolytically cleaved from delta peptide. The majority of delta peptide remains cell-associated, only being released extracellularly at a far slower rate and in lower amounts than sGP [21]. It has been noted that delta peptide reduces the binding of EBOV GP1 to its receptor by interacting with or blocking interactions with host cell attachment factors [15]. This effect is less pronounced in Reston virus (RESTV) than in ebolaviruses that are more virulent in humans. However, the mechanism that limits superinfection of already infected cells has not been elucidated.

Proteins of a number of viruses have been shown to have properties lytic or toxic for host cell membranes, but only one group of viruses, the rotaviruses, has been shown to produce an independent, non-structural protein that serves as a cytotoxin or entertoxin, namely NSP4 [22]. While a number of viroporins, virally-encoded proteins that modify host cell ion permeability, have been described [23,24], NSP4 was the first viral toxin discovered [25,26]. The mechanism of NSP4 action, and the regions of the protein responsible for its toxic properties have been identified [27,28,29]. They involve amphipathic helices capable of inserting into cell membranes, and generating holes or pores in the membranes of infected cells [30]. The enterotoxic effect of NSP4, which requires a multimerization domain [31], is directly responsible for the profuse diarrhea that is characteristic of rotavirus infection of the intestinal tract of young children.

In the course of examining the peptide sequences of all proteins encoded by the West African EBOV of 2014, a number of novel mutations were noted. Some of these mutations drew closer attention to the peptide region where full length GP and sGP/delta peptide are produced from overlapping reading frames. We describe here the discovery of a potential lytic peptide motif in delta peptide with high similarity to peptides known to serve as membrane-destabilizing toxins, such as the NSP4 toxin of rotavirus.

## 2. Materials and Methods

### 2.1. Sequence Information

The following virus strains, used in the sequence comparisons, the gene listing, and their corresponding GENBANK reference listing, are: HIV-1 isolate HXB2 (GP), [GENBANK K03455]; Rotavirus isolate ST3 (NSP4), (GENBANK U59110); Ebola virus 2014, EBOV/SLE14-EM95, (sGP), (GENBANK KM034550); Ebola virus [2], EBOV/Yam76-May, (sGP), (GENBANK NC_002549); Sudan virus, SUDV/Gul-808892, (sGP), (GENBANK NC_006432); Bundibugyo virus, BDBV/But-811250, (sGP), (GENBANK NC_014373); Reston virus, RESTV/Phi89-Pen, (sGP), (GENBANK NC_004161); Tai Forest virus, TAFV/Pau94-CI, (sGP), (GENBANK NC_014372); and Lloviu virus, LLOV/Ast-03-Bat, (GP1), (GENBANK NC_016144).

### 2.2. Computational and Graphic Methods

Sequences in GP and sGP at and beyond the RNA editing site were located by the corresponding notations in the GENBANK listing for that site, and the KK site in GP1 that corresponds to that polyadenine site. Sequences for delta peptide were located within the sGP or, in the case of the putative delta peptide of Lloviu virus, within the GENBANK listing for GP1 that is actually that for sGP, by locating the canonical RXRR endoproteolytic site in sGP and including all amino acids downstream of that site as delta peptide for each virus, and also confirmed by reference to the alignment in Radoshitzky *et al.* [15]. Divergent sequences were aligned by clustal X [32] followed by manual adjustment taking into account not only the peptide sequence but also the corresponding nucleotide sequence. Online tools were used to determine Kyte-Doolittle hydrophobicity (http://web.expasy.org/protscale/) [33], helical wheel and hydrophobic moment representations [34]. The MPEx applet was used to generate values for interfacial hydrophobicity [35]. Three-dimensional modeling utilized the PyMol graphics program [36], coupled with the use of Microsoft Powerpoint and Adobe Photoshop and Illustrator for graphic arrangement and enhancement.

### 2.3. Modeling

Structural modeling was performed via a consensus of models, as performed previously in our previous peptide modeling studies [37,38,39]. PredictProtein (2013 release) was heavily weighted in developing a proposed peptide structure [40], coupled with the reinforcing visualization of a highly amphipathic helix in the presence of a lipid environment. In this instance modeling was additionally informed by the MPEx applet for interfacial hydrophobicity [35] and prior biophysical studies of peptides with similar sequence patterns to delta peptide, such as LLP-1 of HIV-1 [41].

## 3. Results

### 3.1. Delta Peptide is Differentially Conserved amongst Otherwise Disparate Ebolavirus Glycoproteins

In the course of examining the sequence information publicly available for the West African isolates of EBOV from 2014 [42,43], to assess the impact of new mutations, one of us (WRG) examined the variable region of GP (a genome-wide analysis of unique mutations evident in the 2014 outbreak will be presented elsewhere). It was observed that five mutations in GP1, relative to the 1976 reference sequence, are in the region of the protein encoded by the portion of the mRNA after the RNA editing site. He therefore checked to see if any of the five post-editing site mutations in full-length GP (four unique to 2014 isolates) resulted in amino acid substitutions in sGP, which is encoded in a different reading frame. In only three instances out of five is the mutation in GP accompanied by a mutation in sGP as well. To test for conservation in the post-editing site amino acid sequences, the corresponding region of the ebolavirus Tai Forest virus (TAFV) was used as an exemplar of a distantly related sequence. An alignment of the reference Ebola strain EBOV/Yam76-May with the 2014 sequence EBOV-SLE14-EM95 and Tai Forest (TAFV/Pun94-CI) is shown for both GP and sGP downstream of the RNA editing site (Table 1). Overall, it can be seen that conservation between the EBOV and TAFV in the sGP sequence is higher (34/70 [49%] conserved amino acids *vs.* 19/70 [27%]) than in the corresponding region of full length GP. The difference in conservation between the sGP and GP sequences in this region is significant (*p* = 0.014) by Fishers’ exact test.

The RXRR tetrapeptide, which is associated with cleavage of sGP by furin or furin-like endoproteases to yield the larger secreted molecule with high similarity to GP and the largely cell-associated delta peptide, is conserved among ebolaviruses and the putative delta peptide of cuevovirus LLOV [21]. The amino acid sequence in sGP after the canonical RXRR tetrapeptide is also highly conserved. Delta peptides of EBOV and TAFV share 50% identical and 63% chemically similar amino acids. This level of conservation was unexpectedly high, given that EBOV and TAFV are estimated to have diverged from one another a minimum of 2600 years ago and the low conservation in full length GP beyond the RNA editing site among the various ebolavirus species [44].

**Table 1:** Differential conservation in the Full Length Ebola virus glycoprotein *versus* the secreted glycoprotein after the RNA Editing Site *.

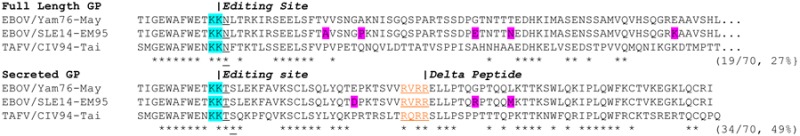

* Shown is a sequence alignment beginning at amino acid 284 of GP1 of the EBOV/Yam76-May reference strain through amino acid 364, the region where the unique sequences of GP1 and sGP are produced from overlapping reading frames. The corresponding regions of EBOV/Yam76-May, the 2014 Cluster 1 sequence EBOV/SLE14-EM95, and the Tai Forest distant outlier sequence TAFV/CIV-94-Tai are aligned by Clustal X [32], confirming their colinearity over this region. The KK dipeptide shown in teal corresponds to the RNA edit site. The third known glycoprotein product ssGP is not shown. ssGP contains only three amino acids beyond the RNA editing site, which are KPH in both EBOVs and TAFV. Amino acid substitutions in the 2014 virus, relative to the 1976 reference strain are shown in magenta; all but the G to R change in delta peptide (also present in 2007 isolates) are unique to the 2014 sequence set of virus isolates. The furin-endoproteolytic site in sGP is in red and underlined, and used as the site to cleave sGP to yield delta peptide from its carboxy-terminal end. Asterisks below the sequence indicate amino acids conserved between all three sequences. The numbers in parentheses at the end of the listing for GP1 and sGP are the number of conserved amino acids in the unique sequences for each.

**Table 2:** Alignment of Membrane Interfacing or Membrane-Spanning Viral Peptide Sequences *.

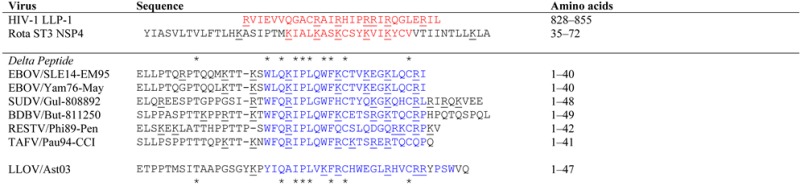

* Shown is an alignment by Clustal X [32] and manual adjustment of lysin sequence motifs in viral lytic peptides and delta peptides of ebolaviruses and a cuevovirus. The first two sequences are of proven lytic sequence motifs present as LLP-1 of HIV-1 glycoprotein, and within the NSP4 protein sequence of Rotavirus strain ST3. The lytic sequence motifs are underlined in red. Proposed membrane-interactive motifs for delta peptides are depicted in blue. In each sequence lysine (K) or arginine (R) residues are underlined to illustrate their alignment across the sequence set, or lack thereof. Asterisks above the delta peptide sequences mark those amino acids that are constantly conserved throughout ebolavirus species of delta peptide, while the asterisks below the delta peptide sequences mark those amino acids that are constantly conserved both in ebolavirus species and in the more distantly related cuevavirus.

### 3.2. Identification of a Lytic Sequence Motif in Ebolavirus Delta Peptide

From previous experience in recognizing patterns of alpha-helical heptad repeats from earlier modeling of the fusion/entry proteins of HIV-1, Ebola, and Lassa fever viruses [37,38,39,45], a 3/4/7 repeat pattern of positively charged amino acids (lysine or arginine) was noted in juxtaposition with a similar repeat pattern in hydrophobic amino acids. A high frequency of amino acids with a propensity to form alpha helices (E, Q, A, L, W, F and K) reinforced the pattern.While the conserved cysteines could initially cause one to consider a conserved disulfide loop structure, the fact that delta peptide is retained in the cell raised the possibility that a disulfide loop may not be favored over the formation of an amphipathic helix. A key finding was the prominent 3/4/7 lysine/arginine repeat, which is highly reminiscent of other viral proteins of known function, namely lytic viroporins. The delta peptide of EBOV shares a lytic sequence motif with known viroporins, rotavirus NSP4 [30] and HIV-1 lentivirus lytic peptide 1 (LLP-1; Table 2) [41].

**Figure 1:**
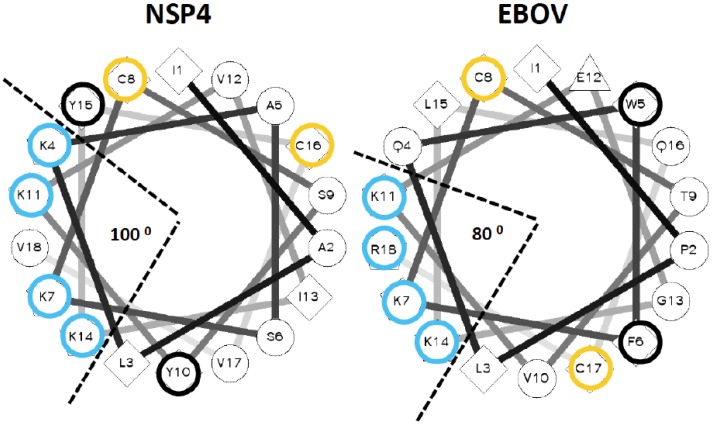
Helical wheel projections for the lytic peptide of NSP4 protein of Rotavirus and the carboxy-terminal half of Ebola virus delta peptide. An online utility was used to project NSP4 and carboxyl terminal amino acids of EBOV delta peptide as an alpha helix [34]. The applet also computes the value and angular direction of hydrophobic moment of each peptide. For emphasis, lysine and arginine residues are circled in blue, cysteine residues in yellow, and aromatic residues in black.

To confirm that the carboxyl terminal sequence of the EBOV delta peptide can be modeled as an amphipathic alpha helix displaying an array of lysine or arginine residues in a 3/4/7 spacing on one aspect, and hydrophobic amino acids on the opposite aspect, the EBOV delta peptide was visualized as a helical wheel projection (Figure 1). The helical wheel representation plots the sequence of amino acids that make up an alpha helix with an angle of 100 degrees between consecutive amino acids. Comparable regions of NSP4 and EBOV delta peptide displayed on a helical wheel projection for each show that in both cases, an aromatic amino acid and a potentially acylated cysteine lie on either side of the helix. In both cases, lysine or arginine residues are predominantly clustered in an array of positively charged hydrophilic residues on one aspect of the helix, subtending an angle of narrow aspect of the helix of only 80–100 degrees. The putative delta peptide alpha helix bears similarity to other classical cationic amphipathic alpha helices that are pore forming (*i.e.*, melittin) [46], but is distinct from not pore forming amphipathic helices, such as Sar1p [47].

We next tested the propensity of the EBOV delta peptide to interact with lipid membranes by the Wimley-White algorithm [35]. The Wimley-White interfacial hydrophobicity scale (WWIHS) is an experimentally determined hydrophobicity scale that predicts regions of proteins that may interface with membranes. Despite the short peptide sequence compromising a test that uses a 10-amino acid window of comparison, EBOV delta peptide had a positive score of 3.66. Much of this is due to the presence of a number of aromatic amino acids in the sequence which greatly favor partition into lipid bilayers [48]. The finding of a peptide sequence with propensity to be membrane-associated simultaneously may explain the retention of delta peptide in cellular membranes after release of the bulk of sGP, and also provides a clear functional significance for the carboxy-terminal 20 amino acids of delta peptide as a viroporin that affects biogenesis of surface proteins.

### 3.3. Delta Peptides of Less Pathogenic Ebolaviruses Have Less Focal Display of Positive Charges

Both length and sequence polymorphisms are evident amongst filoviral delta peptides. However, there are 11 identical amino acids out of 40 shared by all ebolaviruse delta peptides, nine of which are hydrophobic residues (Table 2). The delta peptides of all ebolaviruses contain a conserved region (aa 18 to 38 in EBOV delta peptide) in which 9/21 amino acids are identical (Table 2, sequences in blue). When the distantly related Lloviu virus (LLOV) is included, there are eight identical amino acids among all filoviral delta peptides, seven of them hydrophobic. While the specific positions vary among the delta peptide species, all EBOV and LLOV sequences are enriched in basic amino acids, arginine, lysine and histidine, toward their carboxy-terminal ends. Delta peptides of some ebolaviruses have unique carboxy-terminal extensions (Table 2). The carboxyl terminus of Sudan virus (SUDV) contains three positively charged amino acids (two arginines, one lysine) and two negatively charged amino acids (two glutamic acids). The carboxyl terminal extension of the BDBV delta peptide contains three glutamines, while that of the LLOV contains two aromatic amino acids (tyrosine and tryptophan).

**Figure 2:**
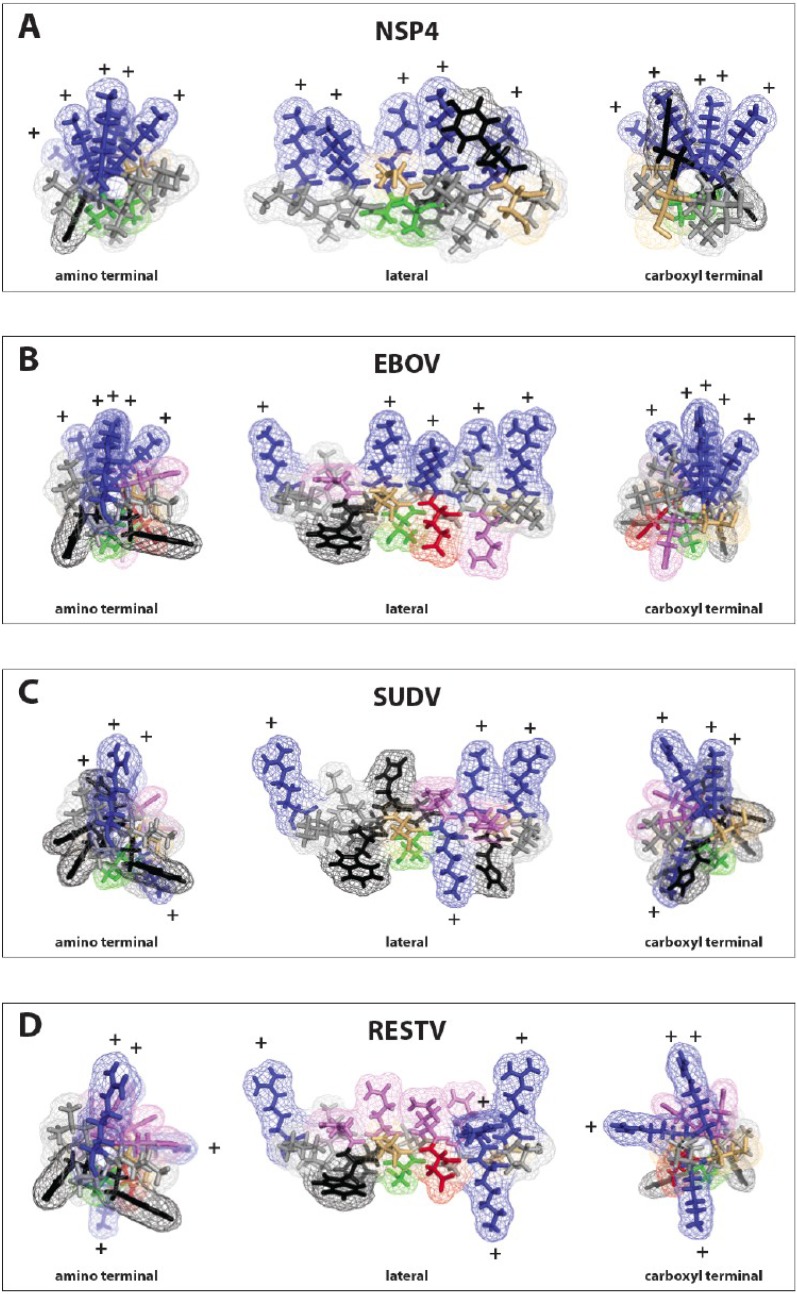
Modeling of the lysin motifs of NSP4 and filovirus delta peptides. The PyMol graphics program [36] was used to model the viroporins NSP4 of rotavirus and the lysin motifs in the carboxy-terminal regions of delta peptides from three different ebolavirus species, EBOV, SUDV and the RESTV as alpha helices. (**A**) rotavirus NSP4; (**B**) EBOV delta peptide; (**C**) SUDV delta peptide; (**D**) RESTV delta peptide. To the left is a projection looking up the helical axis from the amino terminal end of the peptide. To the right is a projection for each peptide from the carboxy-terminal end of the peptide. In the center of each panel is the peptide projection viewed from the side. In all cases, the direction of the maximum hydrophobicity (hydrophobic moment) is downward. Both NSP4 and delta peptide of EBOV project all of their basic (blue) amino acids upwards, while for SUDV and RESV the basic amino acids are more angularly distributed about the axis of the helix.

The delta peptide lysin motif found in EBOV is highly similar to that of NSP4, whether looked at from the aspect that shows the array of lysine residues at the top and hydrophobic residues towards the bottom, or viewed up the axis the helix from the cytoplasmic carboxy-terminus (Figure 2). Both peptides are markedly amphipathic, with a hydrophobic moment oriented in both cases away from the clustered positively charged array, with a value of 3.68 for the lytic NSP4 peptide and 4.16 for the conserved lytic peptide motif in the carboxy-terminus of EBOV delta peptide (Table 3). LLP-1 also has a very high hydrophobic moment of 7.06 confirming this as a trait of viral lytic peptides. SUDV and BDBV delta peptides show marked hydrophobic moments of their conserved lytic peptide motifs (Table 3), although the subtended angles of the arginines and lysines are greater than that of the EBOV delta peptide (80° *versus* 180° and 160°, respectively). When compared with the carboxy-terminal peptide sequences of EBOV, delta peptides from RESTV (Figure 2) and TAFV (not shown) still present multiple lysine or arginine residues in their corresponding delta peptide sequences, but do not as neatly fall into a 3.4.7 periodicity of the lysine or arginine residues, which is reflected in the higher values for subtended angles (Table 3). Whether or not the smaller subtended angle of the positively charged amino acids in EBOV delta peptide confers a greater lytic potential than other delta peptides requires experimental verification. All predicted ebolavirus viroporin sequences have a strongly positive score for Wimley-White Interfacial hydrophobicity (WWIHS), predicting membrane interaction. From the low or nil values for NSP4, LLP-1 or LLOV delta peptide it is clear this is not a prerequisite for lytic peptides. Perhaps the amplitude of the hydrophobic moment and the linearity with which positively charged residues are displayed on the helix may have better predictive value.

All the filovirus delta peptides including those of LLOV show high hydrophobic moments. With such high values for hydrophobic moment, entropic considerations drive these sequences strongly towards forming alpha helices stacking hydrophobic resides in what has been called a “leucine zipper-like” motif, but may actually involve any of a number of hydrophobic amino acid side chains. Indeed the helical projections of delta peptide are much more highly amphipathic than the heptad repeats consistently identified as the structural core of the GP2 proteins of Class 1 fusion/entry glycoproteins. When coupled with the positive WWIHS scores, this engenders confidence in the structural prediction of an alpha helix for the carboxy-terminal 20–25 amino acids of filovirus delta peptides.

### 3.4. Model for Ebola Virus Delta Peptide

**Figure 3:**
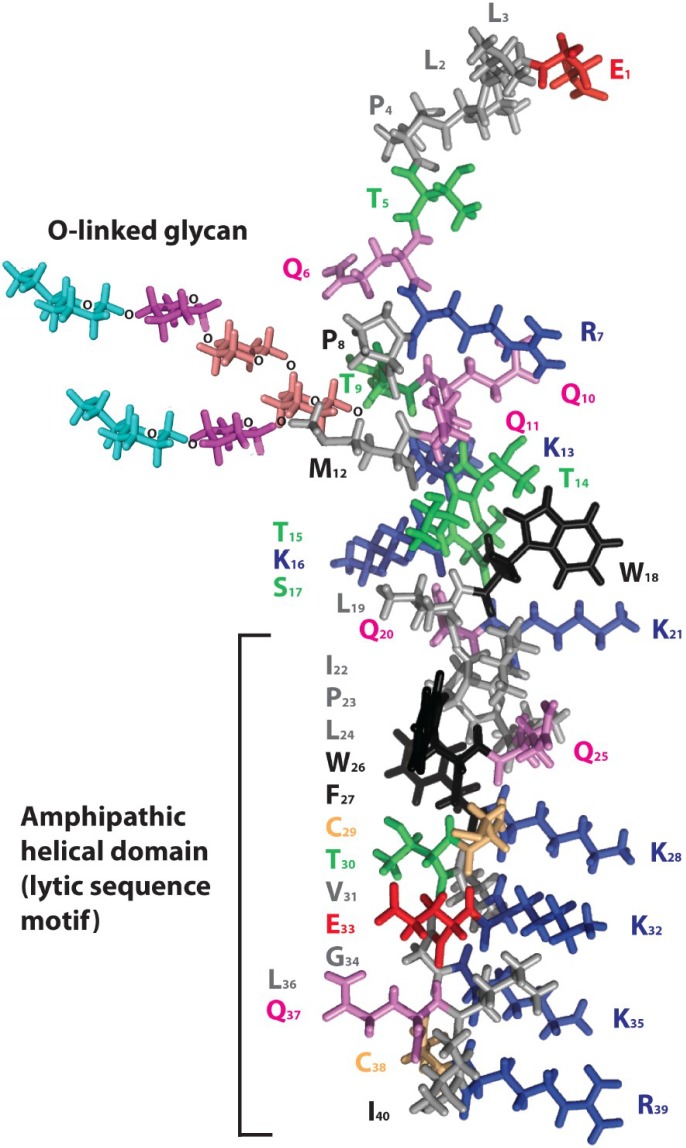
Model for Ebola virus delta peptide. A model of the 40 amino acid peptide sequence of Ebola virus 2014 (EBOV/SLE14-EM95) delta peptide was rendered in PyMol [36] using secondary structural predictions from PredictProtein [40] for the amino terminal half. The carboxyl terminal half of the delta peptide is depicted as an amphipathic alpha helix to emphasize the alignment of positively charged amino acids (lysine and arginine, blue). The carboxyl terminus may not form an alpha helix in a potential alternative configuration of delta peptide in which cysteine 29 and 38 (colored gold) are disulfide-bonded. Aromatic amino acids (tyrosine and tryptophan) are colored black, serine and threonines are colored green, glutamine is colored violet, and the negatively charged amino acid glutamic acid is colored red. The adduct sugar moiety indicates the site of O-glycosylation of delta peptide at threonine 9 of the peptide; the innermost N-acetyl galactosamine is shown in salmon, galactose in purple, and the terminal N-acetyl neuraminic (sialic) acid in cyan. A potential additional O-linked glycan is present at threonine 5.

The observation that the carboxyl terminus of EBOV delta peptide has a putative lytic sequence motif has led to developing a model over its entire 40 amino acid length. Methods similar to those previously employed to first accurately model the fusion and entry proteins of HIV-1, Rous sarcoma virus, Ebola virus, and the arenaviruses, prior to the later structural confirmation of the models by X-ray crystallography, were used [37,38,39]. The model, a consensus of a number of predictive programs, proposes that the leading amino acids of the delta peptide initially have a helical structure, followed by a more disordered region highly variable in sequence but with a constant O-glycosidic adduct at Threonine 9, and then settling into a helical array for the carboxy-terminal half of the protein, as shown in a PyMol computer projection (Figure 3).

Structural studies using NMR or other methods are required to define the precise structure of EBOV delta peptides. In forming the consensus structure, the predictive results from the newest version of ProteinPredict, with a 75% predictive value for known structures, were more heavily weighted. In short peptides, there can also be some plasticity in structure depending on environment, as demonstrated for other small molecular weight peptide toxins [49]. In an aqueous non-reducing compartment, the carboxy-terminal region may form a disulfide closed loop structure and be a less ordered structure. The carboxy-terminal region of delta peptide also contains a conserved proline residue. Prolines have been noted in other lytic peptides, such as LLP-1 [41], and may interrupt alpha helix formation. However, in a lipid–rich or reducing environment, in which disulfide bonds are less stable, the two cysteines in the carboxy-terminal region may not be hydrogen bonded and acylation of cysteines may occur. Free energy considerations suggest that an amphipathic helix may be favored after membrane interaction of delta peptide. The O-linked glycoside is modeled as a mature bi-antennary structure terminating in sialic acid. While the glycoside adduct may be shorter, this size is consistent with the significant effect glycosylation has on mobility of delta peptide in SDS-PAGE, doubling its apparent molecular weight [17], and the fact that the peptide reaches the trans-Golgi where both furin and glycosyl transferases for terminal sugars are located.

**Table 3:** Wimley-White interfacial hydrophobicity scale scores, hydrophobic moment, and angle subtended by arginine and lysine residues for NSP4, LLP-1, and delta peptides of ebolaviruses and cuevovirus *.

Peptide	Sequence	WWIHS Score	Hydrophobic Moment	Subtended Angle Arginines/Lysines
**Viral lytic peptides**				
LLP-1	VVQGACRAIRHIPRRIRQGLERI	0	7.06 @ 53°	180°
NSP4	PTMKIALKASKCSYKVIKYCVV	0	3.68 @ 132°	100°
**ebolaviruses**				
EBOV/SLE14-EM95	WLQKIPLQWFKCTVKEGKLQCRI	3.66	4.16 @ 95°	80°
EBOV/Yam76-May	WLQKIPLQWFKCTVKEGKLQCRI	3.66	4.16 @ 95°	80°
SUDV/Gul-808892	WFQRIPLGWFHCTYQKGKQHCRL	4.98	4.14 @ 100°	180°
BDBV/But-811250	WFQRIPLQWFKCETSRGKTQCRP	4.41	3.02 @ 79°	160°
TAFV/Pau94-CCI	WFQRIPLQWFQCSLQDGQRKCRP	4.41	3.33 @ 52°	160°
RESTV/Phi89-Pen	WFQRIPLQWFRCKTSRERTQCQP	4.41	3.53 @ 126°	180°
**cuevovirus**				
LLOV/Ast03	YIQAIPLVKFRCHWEGLRHVCRRYPSW	1.60	5.92 @ 147°	200°

* Shown are computational analyses of lysin sequence motifs in viral lytic peptides and delta peptides of representative ebolaviruses and a cuevovirus. The MPEx applet, which employes the Wimley-White interfacial hydrophobicity scale (WWIHS) was used to generate values for membrane interfacial hydrophobicity [35]. The hydrophobic moment and subtended angles of lysines and arginines was determined using a helical wheel projection applet [34].

### 3.5. Hypothetical Mechanism by Which EBOV Delta Peptide May Generate Pores within Target Membranes

**Figure 4:**
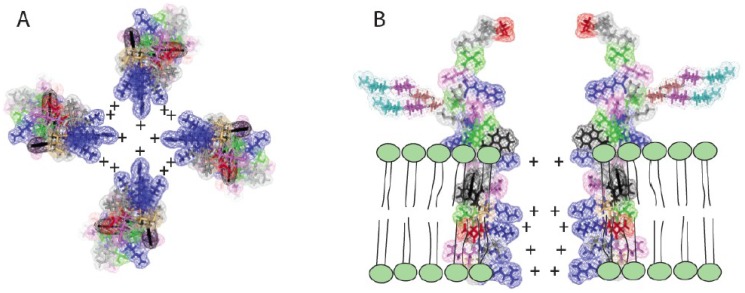
Effect of Ebola virus delta peptide multimerization in the lipid bilayer of cellular membranes. (**A**) A tetramer of delta peptides with the positively charged basic amino acids projecting toward the center of the tetramer can form a pore of the approximate diameter of an alpha helix of approximately 7 to 15 Angstroms. Such a pore, especially when lined with positively charged amino acids, will form an ion channel through which anions such as chlorine may readily pass out of the cell. Such chloride flux is associated with diarrhea and dehydration in humans; (**B**) The potential of EBOV delta peptide to function as a viroporin with a positively charged channel in the target membrane is illustrated in cross section.

In experimental systems with other cationic peptide toxins, lower concentrations of toxin produce ion channels, whereas at higher concentrations permitting formation of multimers the toxins produce pores. In EBOV infected cells, sGP, and, thereby, delta peptide, are made in great excess over full-length GP [50], so it follows that high concentrations of delta peptide would accumulate late in infection, possibly generating an accumulation of pores. The hypothetical mechanism of generating pores within target membranes is illustrated in Figure 4. Within a membrane environment a tetramer of delta peptide molecules in which the hydrophilic positively charged residues are directed inward towards one another, while the exterior hydrophobic face associates with the lipid environment (Figure 4A). Such aggregation and association of the peptide complex with the lipid bilayer may explain how the delta peptide tends to be retained in the cell and in greater amounts than the sGP that is cleaved from it and secreted.

In Panel 4B, a section of the hypothetical viroporin, utilizing the entire delta peptide model is illustrated showing its position in the membrane. Adjacent to the outer lamella of the bilayer, aromatic amino acids (shown in black outline) effect targeting to the lipid environment, and retention of delta peptide in the membrane after cleavage and secretion of sGP. It is this proposed structure, which may accumulate in large numbers in cell membranes, that is projected to act potentially as a cationic pore and toxin creating cumulative efflux of cell contents, both ultimately creating the diarrheal effect and killing the infected cell. Besides toroidal pore formation, other mechanisms by which amphipathic peptides can produce of membrane permeabilization have been described [51,52]. Alternate mechanisms may apply if delta peptide forms a structure in which the carboxyl terminus does not form an alpha helix and the two cysteines present are linked by a disulfide bond. Regardless of the mechanism(s), lipid vesicles or structures resembling virus-like particles (VLP) may be created from the cell membrane that carry the membrane associated delta peptides to other uninfected cells, multiplying a toxic effect in the affected tissue or throughout the body.

## 4. Discussion

Unexpectedly, a peptide motif has been discovered in the carboxy-terminal 20 amino acids of the Ebola virus delta peptide similar to that of known viroporins, such as the rotavirus NSP4 and HIV LLP-1. This is the same region previously found, by alanine-scanning mutagenesis, to be responsible for the reduction in GP1 binding in infected cells [15]. There is correspondence, therefore, between this structural motif and a functional region of delta peptide. These findings suggest that the delta peptide potentially accumulates in cells of the intestinal and hepatic system during EBOV infection and acts as a potent lysin and cytotoxin. If confirmed, delta peptide may prove to be an important virulence factor in EVD.

Not all linear displays of positively charged amino acids in an amphipathic helix would necessarily qualify as candidates for toxins. Such concentrations of basic amino acids are common in proteins interacting with nucleic acid [53]. However, in this case, sGP and delta peptide are already known to interact with, and in the case of delta peptide retained in, cellular membranes while interacting with or blocking interactions with host cell attachment factors [15,21]. The mechanism by which the majority of delta peptide remains cell-associated has not been explained. There is precedence of a cationic amphipathic helix in the pestivirus envelope protein Erns serving as a membrane anchor [54,55]. Furthermore, this raises the index of probability of delta peptide acting as a viroporin with a membrane-lytic effect to a high level. While the greatest molecular parallel between known toxins and viroporins is with virulent EBOV, the molecular motif with enrichment in basic amino acids and high hydrophobic moment extends to all species of ebolaviruses and to the not yet isolated LLOV virus as well.

Phylogenetic analysis using molecular clock computations, and the discovery of portions of filovirus genomes in fossils, have given various estimates of the origin of filoviruses from the end of the last Ice Age over 10,000 years ago, to tens of millions of years ago in the Miocene [44,56]. It is likely that these viruses have circulated in the wild for eons. The high conservation of the filovirus glycoprotein gene after the RNA editing site including the coding sequence for delta peptide indicates that, whatever the function(s) of delta peptide, there is a substantial premium on preservation of that function in the natural host(s) of these viruses.

Direct experimental approaches to work with delta peptide have been very limited. Radoshitsky and colleagues used peptide dimerized with Fc fragments and added externally to cells, to demonstrate its ability to interact with or block interactions with host cell attachment factors [15]. No direct toxic effect on cells was noted. However, externally applied peptide may be very limited in potency, since its normal mode of expression is to be synthesized within the cell as a part of sGP, probably temporarily anchoring sGP in membranes of the endoplasmic reticulum and Golgi, and being dimerized indirectly via the dimerization of sGP. Reproducing the action that the peptide has *in situ* may require extensive development of appropriate systems. However, the molecular parallels in the pattern of delta peptide to known viroporins, together with the current emergency status of EBOV infection in West Africa, compelled us to make our observations public at this very early stage of discovery.

Genetic constructs used in animals, creating chimerae of the genomes of different species of ebolavirus implicate products of the GP gene as partly, but not entirely responsible for virulence. An ebolavirus genome containing only the GP gene for RESTV, against a background of all other six genes of the virus from EBOV, had reduced virulence [57]. However, the mechanism(s) or region of GP that correlates with virulence has not yet been determined. Some members of the *Filoviridae* are also capable of causing a transient immunosuppression during acute infection, compromising the immune response and recovery of the host [58]. Multiple mechanisms of immune suppression include the elaboration of large amounts of secreted EBOV glycoprotein to serve as a decoy diverting antiviral antibodies from neutralizing the virus or eliminating infected cells [59], antagonism of human interferon by VP35 [60], an immunosuppressive region of GP2 homologous to that seen in retroviruses [61], and immune activation and increased vascular permeability [62]. Except for role of sGP as a decoy molecule, and the anti-interferon effect of VP35, no pathogenic mechanisms for Ebola or Marburg diseases have been elucidated. The studies presented here should focus attention on the highly conserved delta peptide as a potential cytolysin or enterotoxin involved in virulence of ebolaviruses.

Ion channel proteins and peptides are broadly distributed in nature and function through a wide variety of molecular mechanisms. Many bacterocins, bacterial toxins, insect toxins such as bee venom, and human defensins, either possess ion channel activity or interact directly with cellular ion channels. The cationic ion channels typical of viroporins, such as NSP4, and, now potentially EBOV as well, may function in several ways. One, at low density, would be a small ion channel of low potency, and at higher densities, an actual membrane pore allowing cellular contents to exit freely across the damaged membrane. Cytolysis by paramyxoviruses via a fusogenic mechanism, such as by Newcastle disease virus, follows this dynamic, with little or no direct membrane damage at low virus-target ratios, low temperature or in the presence of divalent cations, but massive cytolysis and protein release in high virus dosage at 37C and when divalent cations are omitted from the media [63,64]. Protein toxins and viroporins may also activate cellular mechanisms such as phospholipases.

Delta peptide is likely cleaved in the trans-Golgi [65], and is consistently O-glycosylated. The delta peptide glycan may terminate in sialic acid, which is also added in the trans-Golgi. Because the peptide sequence also contains one or a few glutamic acid residues, it may be self-gated as an ion channel in a manner similar to the M2 viroporin of influenza virus [66]. A number of experimental approaches are available to test the hypothesis that delta peptide is a viroporin. Synthetic peptides representing the EBOV delta peptide can be tested for lytic function in liposome disruption or vesicle content mixing assays. Other filovirus proteins, such as VP40 [67], are known to possess membrane interactive domains and potentially cooperate with or contribute to delta peptide function. Thus, additional experimental approaches to the natural regulation of delta peptide, potential inhibitors of its putative viroporin function, and its possible interactions with other viral membrane-associated proteins, present a number of avenues in future investigation of filoviral pathogenesis.

## 5. Conclusions

We have described a conserved, amphipathic peptide region of ebolavirus and cuevavirus delta peptide that is enriched in lysine and arginine residues and has a high propensity to form an alpha helix displaying the basic amino acids in a linear array along a narrow aspect. The hydrophobic moment of the carboxy-terminal 20 amino acids of delta peptide coupled with a narrow angle of helical display of positively-charged amino acids, is positively correlated with virulence. Such a molecular motif is characteristic of lytic peptides and previously characterized viroporins. An ion channel peptide with high affinity for membranes is consistent with the known properties of delta peptide associating with cellular membranes and in reducing expression of host membrane proteins such as those that bind EBOV. The discovery of a potential lytic peptide motif in the delta peptide of EBOV, in the same class of cationic pore-forming peptides as NSP4 of rotavirus, when considered together with the large amounts of sGP produced during infection, suggests that delta peptide may contribute to EBOV pathogenesis.
